# Applying a Three-Tier Approach to Address Gaps in Oral Pre-Exposure Prophylaxis Uptake and Continuity in Uganda: A Mixed Methods Approach

**DOI:** 10.9745/GHSP-D-23-00229

**Published:** 2024-04-29

**Authors:** Simon Sensalire, Abel Nkolo, Juliana Nabwire Ssali, Martin Muhire, Augustin Muhwezi, Herbert Kadama

**Affiliations:** a U.S. Agency for International Development Uganda Health Activity, Kampala, Uganda.; b University Research Co., LLC, Washington, DC, USA.; c Ministry of Health, Kampala, Uganda.

## Abstract

**Background:**

We describe a 3-tier approach involving a gap analysis, root cause analysis, and pre-exposure prophylaxis (PrEP) collaborative to understand the gap and identify and address the main barriers to oral PrEP uptake and continuity in Uganda.

**Methods:**

We used a mixed methods design with retrospective, cross-sectional, and prospective components. The gap analysis involved descriptive analysis of PrEP uptake and continuity. The RCA identified the main barriers to initiation and continuity on PrEP among 1,334 clients who declined to start PrEP and 1,266 who missed their appointment but were at risk. The PrEP collaborative tested changes mapped onto specific barriers to optimize the PrEP clinical service delivery. A trend analysis of routinely collected data of the PrEP cascade determined significant shifts and trends in PrEP uptake and continuity.

**Results:**

Only 60% of the high-risk population eligible for PrEP were enrolled, while fewer than 30% of the cumulative number of PrEP users were refilled each quarter. Uncertainty and fear of side effects, perceptions about the harmfulness of the medication, perceived inability to adhere to PrEP, and stigma were the main reasons why clients rejected PrEP. Lack of access to the facility, side effects, pill burden, stigma, perceived low-risk exposure to HIV, and preference of staying at work as opposed to picking up refills affected continuity on PrEP. The collaborative registered statistically significant shifts in PrEP enrollment from 64% to 89% and continuity from 51% to 78% between July 21 and November 22 following the collaborative intervention.

**Conclusions:**

We recommend using a 3-tier approach for other similar implementation contexts to strengthen PrEP programming, given the marked statistical shift in PrEP uptake and continuity. This begins with understanding the gap and barriers to use among clients, followed by mapping interventions to specific barriers through a quality improvement collaborative.

## Abstract (precis)

We introduced a transformative approach consisting of a gap analysis and root cause analysis to understand and address significant gaps in enrollment and continuity on oral pre-exposure prophylaxis (PrEP) and a national quality improvement collaborative to map interventions to address specific barriers in the PrEP cascade.

## Teaser

**Key Findings:**

- A gap analysis showed that both uptake of oral pre-exposure prophylaxis (PrEP) and continuity on PrEP were far below the Uganda national targets.
- A root cause analysis investigated why clients were not willing to start PrEP and revealed that clients had concerns about taking PrEP daily and its side effects, held negative perceptions about PrEP and lacked information, had a low perceived HIV risk, and felt stigma associated with PrEP.
- Clients reported reasons for missing appointments for PrEP or not returning for refills were long distance and transport costs to the health facility, negative experiences of the drug, stigma, forgetfulness of appointment dates, perceived low HIV risk, pill burden, and challenges related to food availability.
- During the collaborative intervention, statistically significant improvements were observed in the PrEP cascade, including PrEP uptake, keeping of appointments, and reinitiation on PrEP.

**Key Implications:**

- Program managers should consider adopting collaborative efforts involving peers to help influence the uptake of PrEP.
- The barriers to initiating and continuity on PrEP are numerous—sociocultural, behavioral, structural, and knowledge factors—and require multilevel and tailored interventions based upon context.

## BACKGROUND

In 2016, Uganda launched oral pre-exposure prophylaxis (PrEP), revising its HIV guidelines to recommend the prescription of PrEP for HIV-negative persons at substantial risk of acquiring HIV, beginning with a few accredited antiretroviral therapy (ART) health facilities.^1^ Various studies have documented populations that are at substantial risk of HIV in Uganda. A study of 46 fishing communities found an HIV prevalence of 22% among fisher folks, and the prevalence among sex workers was estimated at 37% in 2015/2016.^2^^,^^3^ A report estimated HIV prevalence among people who inject drugs at 16.7% in Uganda.^4^ However, less than half of individuals diagnosed with sexually transmitted infections or other markers of HIV risk accept an active offer of PrEP.^2^ The effectiveness of PrEP depends on its acceptability and accessibility as part of a comprehensive HIV prevention package. In the absence of these components, even the most highly efficacious PrEP medication will have little to no impact in reducing HIV infections.

There are many challenges to accessing and adhering to PrEP, as reflected in low levels of PrEP uptake.^5^ Concerns over rates of adherence and continuity have been reported in PrEP care clinical trials and PrEP demonstration projects.^6^ In most parts of sub-Saharan Africa, research has focused mainly on understanding why clients are not taking or adhering to PrEP without evaluating complementary improvement interventions to address these barriers. Therefore, to comprehensively guide the national PrEP program in Uganda, a 3-tier approach was undertaken involving: (1) a gap analysis to determine the current uptake of PrEP against targets for all priority subpopulations, (2) a root cause analysis (RCA) to identify barriers to uptake and continuity on PrEP, and (3) a PrEP quality improvement (QI) collaborative to address quality gaps in the PrEP cascade. The results and key learning would be shared to facilitate the spread of improved performance.^7^ The cascade, which includes conducting a medical evaluation for PrEP eligibility, initiating PrEP, and continuing PrEP services, provided measurable benchmarks from which to evaluate progress in the implementation of the PrEP collaborative and inform the development of interventions to optimize uptake and continuity in PrEP services.

In most parts of sub-Saharan Africa, research has focused mainly on understanding why clients are not taking or adhering to PrEP without evaluating complementary improvement interventions to address these barriers.

In this article, we describe the 3-tier approach to understand the gap and identify and address the main barriers to PrEP uptake and continuity. We show the results from each tier, characterize key steps of the PrEP cascade, and identify correlates of continuity on PrEP. In this article, we define continuity on PrEP as the sustained usage of PrEP for as long as an individual remains in the season of risk of acquiring HIV. This definition excludes clients who have been objectively assessed and found to be no longer at risk of HIV transmission or off PrEP because they are not in a season of risk.

## THE THREE-TIER APPROACH

### Conduct a Gap Analysis to Assess Gaps in Enrollment and Continuity

The gap analysis assessed the gap in enrollment and continuity of clients on PrEP against the set national targets. The targets were based on data from the Ministry of Health (MOH) key population tracker, which collects data on subpopulations prioritized for PrEP from 2019 and 2020, covering 142 health facilities. Client eligibility was determined using the eligibility screening tool. It was projected that by the end of 2021, 260 facilities would be implementing PrEP across the country, with 90,000 clients ever enrolled.^8^ Oral PrEP using tenofovir and emtricitabine as a once-daily pill is highly effective in preventing HIV infection.^9^ Our gap analysis compared the cumulative number of PrEP users that received refills every quarter to a 100% continuity target of all high-risk clients.

### Use a Root Cause Analysis to Identify Factors Influencing Uptake

RCA is a process for identifying the causal factors underlying variations in performance and has been adapted for routine use in health care.^10^ Our RCA was based on the COM-B (capability, opportunity, motivation, behavior) model for behavior change. Capability refers to an individual’s psychological and physical ability to participate in an activity, opportunity refers to external factors that make a behavior possible, and motivation refers to the conscious and unconscious cognitive processes that direct and inspire behavior.^11^

We considered that uptake of PrEP and continuity may be influenced by many factors. Therefore, our RCA aimed at determining client-perceived HIV vulnerability, determining barriers to uptake and continuity on PrEP, and identifying lapses in system-level processes in PrEP-accredited sites. There is a general consensus that RCA uses a “toolbox rather than a single method,” with team-led investigations typically attempting to ascertain the “why” of a given phenomenon or incident.^12^^,^^13^ We asked clients the following questions: what is the main reason why you are not willing to start taking the drug that reduces the risk of getting HIV? and what is the main reason why you missed an appointment or did not come for a refill? The root causes were then determined through Pareto analysis, one of the analytical and problem-solving techniques.^14^ Facility improvement teams were guided by the RCA to test change ideas to address gaps in initiation and continuity. The team used the MOH documentation journey to document the problem being addressed, root causes, change ideas tested to address the problem, and performance trends from implementing the change ideas.

### Implement a Quality Improvement Pre-Exposure Prophylaxis Collaborative to Address Deficiencies

The national PrEP QI collaborative was implemented using the national QI structure to address process deficiencies in PrEP service delivery and anchored on the existing QI structures defined by the National QI Framework & Strategic Plan (2021–2025). The National QI Task Force sets the national QI agenda and coordinates all QI partners. The national and regional QI coaches are health workers with QI experience and support district QI teams. District QI coaches are health providers designated to support facility work improvement teams that comprise key facility staff designated to test and implement change ideas to address process gaps at 257 PrEP sites. These facilities are typically government health centers above health center II and private not-for-profit facilities that have been trained to offer PrEP counseling, prescription, and monitoring services mostly at the ART clinic or HIV prevention clinics.

The national PrEP QI collaborative was implemented using the national QI structure to address process deficiencies in PrEP service delivery.

**FIGURE 1:**
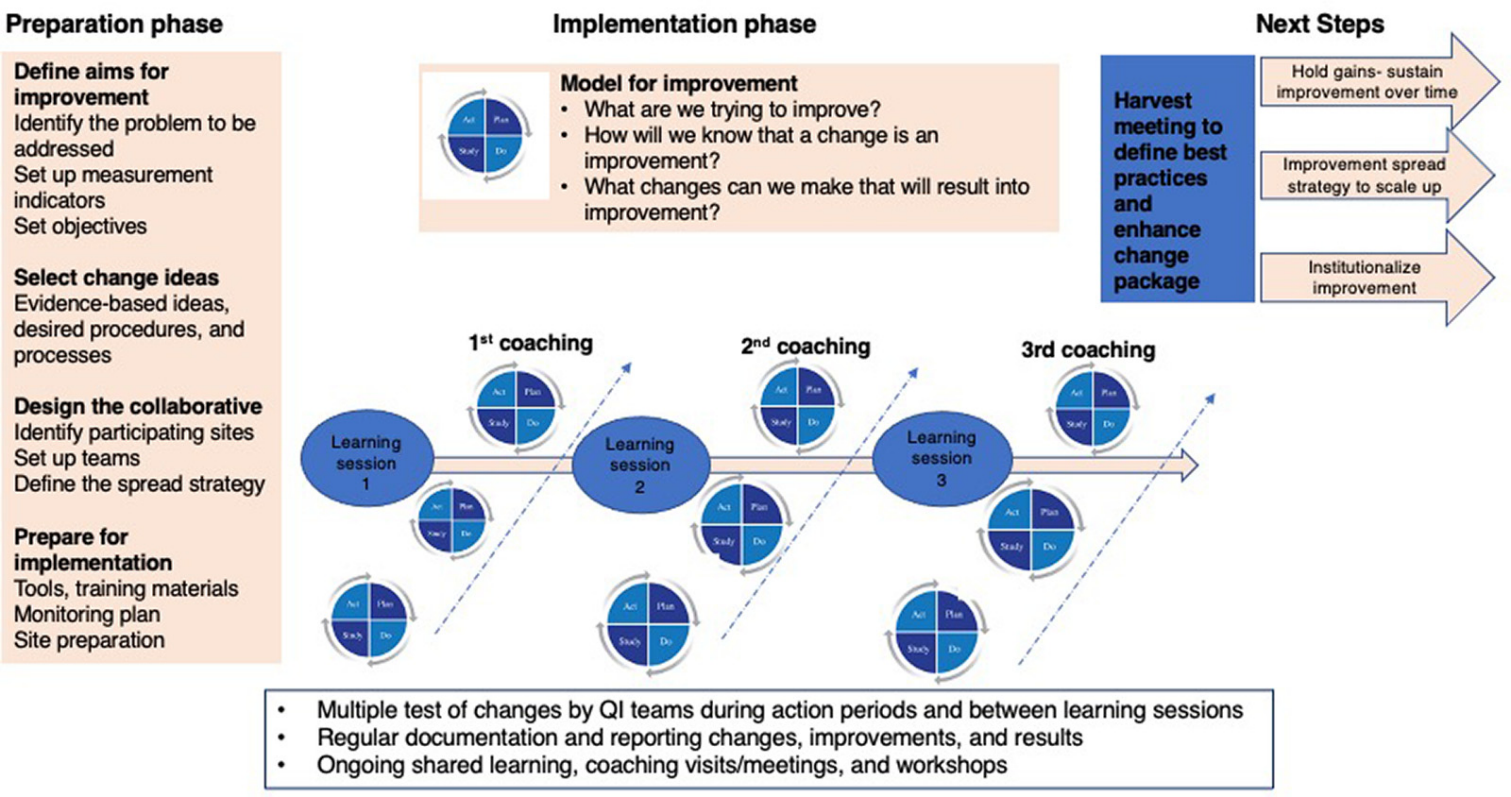
Steps for Preparation, Testing, and Implementation of the PrEP Collaborative Abbreviations: PrEP, pre-exposure prophylaxis; QI, quality improvement. Adapted from Breakthrough Series Model (Institute for Healthcare Improvement, 2003).

The steps taken for preparation, implementation, and testing of the national PrEP collaborative drew from previous best practices in the Ugandan context on QI collaboratives (Figure 1).

#### Preparation

The initial phase of the PrEP collaborative involved developing and testing tools in 8 health facilities in May 2021 before rollout to 257 sites of the collaborative in July 2021. Baseline data collection tools, PrEP continuity tracking tool, coaching guide, and data reporting system were piloted. The tools were used by facility work improvement teams to track improvement activities and for coaches to support the teams.

Implementing partner (IP) technical staff, coaches, and PrEP focal persons were oriented on PrEP technical content, QI basic principles, tools, and guides, followed by developing facility workplans. As part of their routine mandate, IPs routinely supervised coaches who supported intervention sites.

Facility work improvement teams, with the support of MOH-designated coaches, systematically collected baseline data on PrEP screening, initiation, and continuity from 257 sites on the following cascade indicators.

- PrEP uptake: The proportion of PrEP-eligible clients initiated on PrEP, calculated as the number of clients who were started on PrEP divided by the total number who were medically evaluated and found eligible for PrEP. A client was eligible for PrEP in this study if they were screened and found to be exposed to 1 or more risk factors: had vaginal or anal intercourse without condoms with more than 1 partner; had a sex partner with 1 or more HIV risk; had a history of a sexually transmitted infection (based on self-report, lab test, syndromic sexually transmitted infection treatment); had taken post-exposure prophylaxis following a potential exposure to HIV; reported having a sexual partner who was HIV positive and who had not been on ART for at least 6 months, or had inconsistent or unknown adherence.
- Continuity: The proportion of PrEP clients scheduled for appointment that kept it, defined as clients eligible for PrEP presenting for their follow-up appointments divided by the total number of scheduled clients in the reporting period.
- The proportion of PrEP users who missed drug pick-up in the previous month with a documented follow-up outcome. Patients who did not return within 30 days of a scheduled follow-up visit after PrEP initiation were followed up and their status documented (such as either had transferred care to another facility or discontinued PrEP).
- The proportion of PrEP users who were followed up, still at risk of HIV, and were reinitiated.

#### Implementation

In August 2021, MOH-supported QI coaches conducted their first coaching visits to support facility work improvement teams to use data to identify their own PrEP gaps, conduct RCA, and test and implement changes to address the gaps within their contexts.

These multidisciplinary facility QI teams were formed in PrEP-accredited sites to address the root causes within their community contexts to minimize the risk of reoccurrence.^17^ The change ideas to address the root causes were focused heavily on processes of screening and enrollment on PrEP of those at risk, appointment keeping for those still at risk, and returning of those who missed appointments and still at risk (Supplement).

Coaching was facilitated by a guide that details the process of identifying and addressing gaps and documenting improvements by the facility work improvement teams.

Health facilities reported on the progress of the PrEP collaborative indicators through the existing online U.S. Agency for International Development-supported national QI database used to track and monitor the national QI initiative at facility, district, regional, and national levels on a monthly basis. QI coaches used the data to prioritize sites needing additional support and technical assistance.

Each regional IP was supported to conduct at least 2 learning sessions in their respective regions to facilitate cross-learning on how to improve initiation and continuity across the participating health facilities. In addition, 2 national-level learning sessions were conducted by the MOH and U.S. Agency for International Development Uganda Health Activity, spaced 4 months apart to facilitate learnings across the regions. Learning sessions brought together facility QI teams from different facilities to share best practices and results from the implementation of best practices.

### Harvest High-Impact Innovations to Address Gaps

After 12 months of the PrEP QI collaborative, a meeting was organized to systematically synthesize and harvest high-impact innovations for addressing gaps in the PrEP cascades by the facility work improvement teams. The harvesting team included individuals from the MOH; QI focal persons of IPs; above-site partners that are organizations designated to provide support to local partners directly involved in quality improvement activities at the sites; national, regional, and district coaches; and facility work improvement teams from 3–4 best-performing sites in each of the regions.

A meeting was organized to systematically synthesize and harvest high-impact innovations for addressing gaps in the PrEP cascades by the facility work improvement teams.

Facility work improvement teams presented their QI projects (e.g., gap, improvement aim changes that were tested, and results). After the presentations, facility QI teams developed a master list of changes. Documentation journals were used to validate evidence of improvement associated with the given change. The teams were constituted according to the improvement objective (which also represented the PrEP cascade) to evaluate the changes by assigning scores from 1 to 5 (1 being the lowest and 5 the highest) on the basis of evidence from the pilot, relative importance, simplicity, and affordability. The median score was then determined and any changes equal to or above the median were classified as a best practice and further discussed to generate a “how-to guide” that describes in detail how the change was implemented. The outcome of the harvest was a change package (Supplement) that shows the problem, change ideas applied by sites to address the problem to improve uptake and continuity of PrEP, and the magnitude of improvement.

### Address Supply Chain and Stock Management Issues

The MOH and IPs supported health facility teams to maintain adequate stock of PrEP commodities at the health facilities. Tools to address the stock challenges were integrated into QI coaching to support health facilities to properly forecast their needs of the PrEP commodities and limit stock-outs, one of the contributors to discontinuation.

### Support Communication and Social Mobilization Efforts

The MOH, in collaboration with the U.S. Agency for International Development-funded Social Behavior Change Activity, developed robust information, education, and communication materials to support advocacy and social mobilization on PrEP use for HIV prevention. At the community level, the engagement of the existing community health structures and champions, such as peers’ leaders, was critical in creating awareness for service uptake and addressing myths about PrEP.

## METHODS

### Design

We used a mixed methods design with retrospective, cross-sectional, and prospective components, which were nested in an improvement intervention aimed at understanding the gap, its root causes, and how to address them. The gap analysis involved studying national performance against targets, and the cross-sectional study was an RCA based on the COM-B model for behavior change. The retrospective and prospective components involved extracting data from medical records of all consecutive patients screened for PrEP eligibility initiated and retained on PrEP 3 months before and during the PrEP collaborative. Deidentified data were abstracted monthly to assess trends in the PrEP cascade. The integration of 3 distinct methodologies served as the foundation for identifying and understanding the barriers to both uptake and continuity on PrEP. Subsequently, we devised targeted change ideas detailed in Table 1 and the Supplement to directly address the underlying barriers identified among our target population.

**TABLE 1.** Change Ideas Implemented to Address Barriers of PrEP Initiation and Continuity

Specific Problem Being Addressed	Tested Changes
**Best practices for improving PrEP initiation**	
PrEP tools do not have provision for recording PrEP screening	Improvised PrEP screening column in the HTS, antenatal care, postnatal care registers to record “C-SCN” to mean that client was screened for PrEP and “E-SCN” to mean client is eligible for PrEP
Low screening due to low utilization of the PrEP screening tool	Attach PrEP screening tool to every HTS client card before screening is done
No specific person to screen for PrEP eligibility at the HTS point of care	Assign a specific person to screen for PrEP at all HTS point of care
PrEP screening was not done at all entry points	Conduct PrEP screening during other activities of the facility such as Young Child Clinic, antenatal care, and voluntary medical male circumcision
Low PrEP initiation at hot spots	Attach peers to specific hot spots to sensitize clients on PrEP
Lack of awareness about PrEP	Attach satisfied PrEP users to do group and individual education at facility and community
Incomplete documentation of PrEP registers	Form and task a team to review PrEP records for completeness
Low uptake of PrEP	Assign a focal person for PrEP activities Identify skilled and willing staff at entry points to take charge of PrEP initiation
Lack of a system to track clients due for appointment	Use of SMS/phone calls as reminders for clients due for appointment
Incomplete clients record in some PrEP tools	Develop and display a check list of tools required by health workers to complete during PrEP initiation Conduct targeted continuing medical education to new staff/departmental representatives from all entry points on PrEP initiation
Long distance for clients to access services	Conduct targeted outreaches
**Change ideas for improving appointment keeping**	
Lack of a system to track clients due for appointment	Use of SMS/phone calls as reminders for clients due for appointment
Client does not show up for appointment	Attach clients due for appointment to peers
Health workers do not know clients’ locations for follow-up	Develop a locator form and update locations whenever they return to the clinic
Transport challenges by clients	Prioritize hot spots with high numbers of clients to deliver PrEP refills to them
Client stigma while at the facility	Avail PrEP refills at drop-in centers
Clients experience long wait times at the clinic	Conduct PrEP refills during outreaches of other activities in the community Set up a one-stop center at the facility
Patients’ illiteracy on PrEP	Translate information, education, and communication materials in local language
Lack of documentation of follow-up outcomes	Develop and update the locator form Generate a line list of missed appointments Make a phone call to clients who missed appointments
Lack of responsible person to track missed appointments	Assign a health worker and peers to physically follow up missed appointments
Knowledge gap for health workers on filling the appointment register	Hands-on orientation on filling the appointment register
Forgetting to update follow-up outcomes	Same day update of follow-up outcomes

Abbreviations: HTS, HIV testing services; PrEP, pre-exposure prophylaxis; SMS, short message service.

### Study Population and Sample Size

The gap analysis focused on clients who were eligible for PrEP and those initiated on PrEP across PrEP-accredited sites in Uganda, which are health care facilities that have been authorized to provide PrEP services and provide comprehensive care, including ART.

The RCA was conducted among seronegative clients eligible for PrEP but who declined to start and those who did not present for their follow-up appointments. We estimated a sample of 1,334 clients who declined the offer to start PrEP and 1,266 clients who missed their appointment schedule. Using Cochran’s sample size formula, we considered an alpha of 5% and critical value for a 95% confidence interval of 1.96 and precision of 5%. Prevalence (*p*) for the various categories of clients was estimated from previous studies published in the study domain.^14^

The PrEP collaborative focused on clients eligible for PrEP and those initiated on PrEP during the intervention period.

To eliminate selection bias, consecutive sampling was used to select high-risk clients for each of the 3 categories of clients: clients who declined to start PrEP, eligible clients on PrEP who missed appointments, and clients who were reinitiated on PrEP. The study aimed to cover a sizable number of clients in each category to reach saturation effect. Hennink et al. define saturation as the point at which “no additional issues are identified, and the codebook begins to stabilize.”^15^ Given the potential for uncertainty about the point at which saturation is reached, we continued collecting information until the desired sample was obtained, even when there were no further insights about PrEP being documented. In line with Constantinou et al.,^16^ we were preoccupied with having enough data beyond saturation to seek additional objective evidence to bolster programmatic decisions.

### Data Collection, Management, and Quality Assurance

Data used in the gap analysis were extracted from Uganda’s key population tracker. We extracted nonidentifiable data on demographic and service characteristics to identify gaps in service among the key populations using data export tools built within the tracker system.

The RCA was conducted at health facilities as part of the 5As (Assess, Advise, Agree, Assist, Arrange) model used in behavior change. The RCA is institutionalized in our care settings as part of the 5As to routinely determine barriers to care. The main barriers to initiating and continuity on PrEP were obtained during counseling of the client, with more interactive opportunities to weigh in on the main barriers. Eligible clients who declined the offer to start PrEP were then asked as part of the 5As about the main reason why they declined to start PrEP through outreach or phone interviews. Relatedly, those who interrupted PrEP on account of missed appointments or failing to pick drugs were asked the main reason for missing their appointment. Other information collected included the client’s age, category, risk factor, duration on PrEP, and HIV risk perception.

Each unique response was matched with a tally corresponding to the clients mentioning it. When a single barrier was mentioned multiple times by different clients, a tally was marked against all clients mentioning it without having to capture it again. Each unique barrier was then coded and matched with other variables corresponding to each client in Excel. To maintain uniformity across sites, we applied the same procedures for data collection and tools across the PrEP-accredited sites. Health providers were oriented on the procedures and tools for quality control and for standard application across PrEP sites. The RCA tools contained simple and clear instructions for providers to guide the interview and tallying. Data on the PrEP cascade were extracted monthly from HMIS registers by the PrEP focal persons at each facility and submitted electronically throughout the PrEP collaborative.

### Data Analysis

Descriptive analysis using numbers and percentages was conducted to determine gaps in PrEP uptake and continuity and trends in the PrEP cascade following the PrEP collaborative.

The RCA was performed using the Pareto chart. The charts are based on the “80/20” rule, also known as the Pareto Principle, which asserts that 80% of outcomes result from 20% of all causes for any given event. Pareto charts showed the ordered frequency counts of values for the different levels of causes/barriers and identified areas to focus on for improvement. Descriptive statistics illustrated clients’ characteristics using numbers and proportions.

### Ethical Approval

All procedures performed in the intervention study were approved by The AIDS Support Organization institutional research board. Client consent for data abstraction was waived because there was no physical interaction with the client. The data are deidentified and readily available on HIV program platforms.

## RESULTS

### Gap Analysis

Data from the MOH key population tracker for the period 2019 and 2020 showed that only 60% (42,000) of the high-risk population eligible for PrEP at 142 health facilities were enrolled, far below the expected 80% national target. Further analysis of data showed that less than 30% of the cumulative number of PrEP users were refilled every quarter, way below the desired 80% of the national continuity target.

### Root Cause Analysis

Table 2 shows the characteristics of clients who declined to start PrEP and those who missed their appointment schedules. Clients who declined to start PrEP were mostly people aged 15–24 (46%) and 25–34 years (35%). Like their counterparts who declined PrEP, clients who missed appointments were mainly aged 25–34 years (44%).

**TABLE 2.** Characteristics of the Clients Who Declined to Start PrEP and Those Who Missed Appointments

	Clients Who Declined PrEP	Clients Who Interrupted PrEP
	No. (%) (n=1,334)	No. (%) (n=1,266)
Age group, years		
15–24	617 (46.3)	172 (13.6)
25–34	469 (35.2)	379 (29.9)
35–44	184 (13.7)	301 (23.8)
45+	64 (4.8)	414 (32.8)
How likely is it that you could get HIV because you are not using PrEP?		
Not likely at all	254 (19.0)	263 (20.8)
Unlikely	324 (24.3)	214 (16.9)
Likely	390 (29.2)	332 (26.2)
Extremely likely	139 (10.4)	295 (23.3)
Undecided/don’t know	227 (17)	162 (12.8)
How likely is it that your partner will suggest to you to use PrEP during the time of engaging in risky sexual behavior?		
Not likely at all	532 (39.9)	516 (40.8)
Unlikely	313 (23.5)	282 (22.3)
Likely	161 (12.1)	229 (18.1)
Extremely likely	64 (4.8)	71 (5.6)
Undecided/don’t know	264 (19.7)	168 (13.2)

Abbreviation: PrEP, pre-exposure prophylaxis.

#### Perceived Vulnerability and Partner Support

This study assessed clients’ perceived vulnerability to HIV. Among clients who declined to start PrEP, 29% of clients perceived they were likely to get HIV. Almost a similar proportion (26%) who missed their appointment perceived themselves as likely to get HIV because they were not on PrEP.

Among clients who declined to start PrEP, 29% of clients perceived it likely for them to get HIV.

Clients were asked how likely their partner would propose PrEP in high-risk sex. There was low perceived partner support for PrEP in high-risk sex, with 63% of clients who had declined PrEP indicating that their partners would be unlikely to suggest PrEP use in high-risk sex. Similarly, perceived partner support to use PrEP among clients who had missed appointments was low (26%).

#### Risk Factors Among Clients Who Declined the Offer for Pre-Exposure Prophylaxis

**FIGURE 2:**
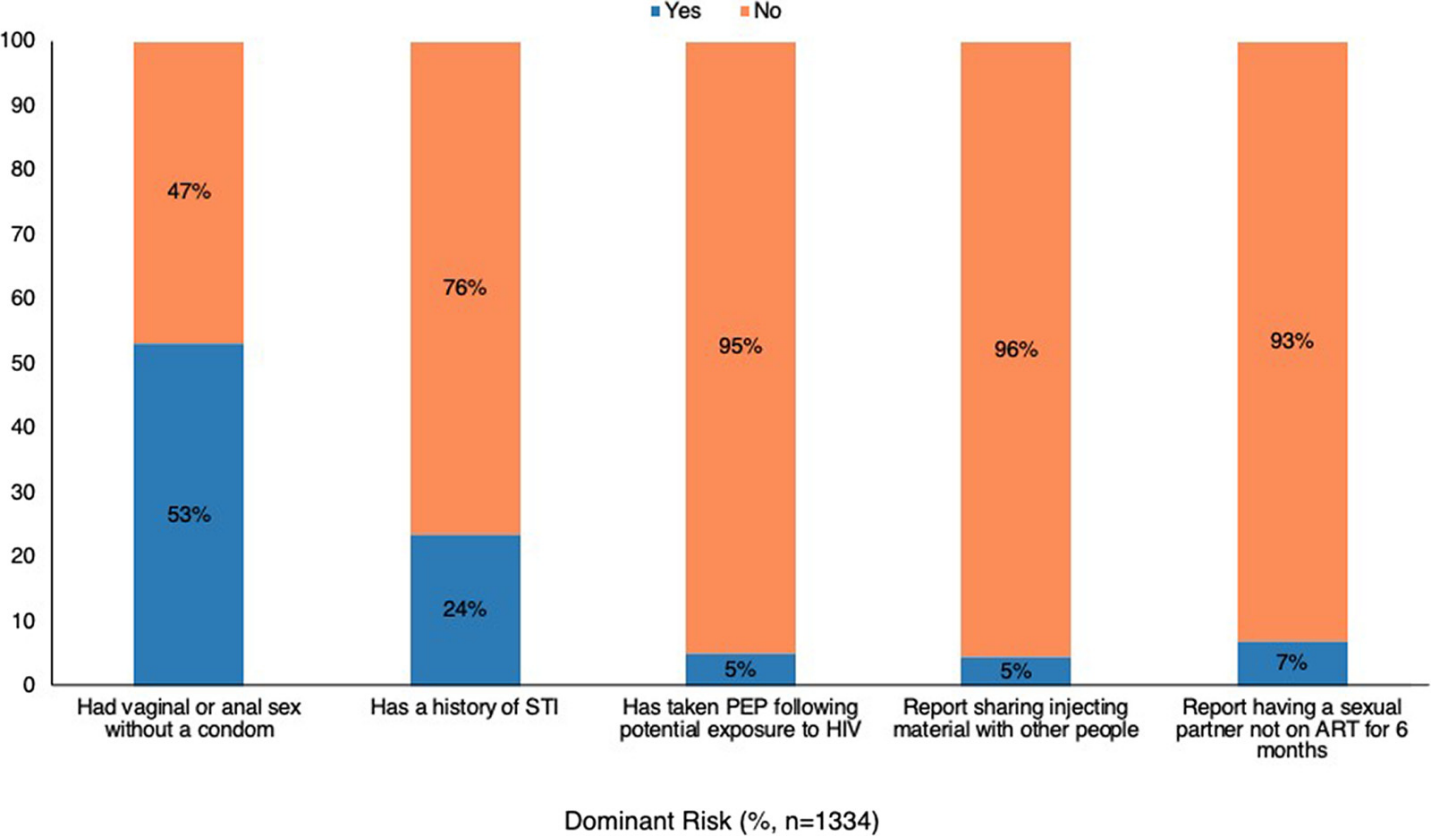
HIV Risk Factors Among Targeted PrEP Clients Abbreviations: ART, antiretroviral therapy; PrEP, pre-exposure prophylaxis; STI, sexually transmitted infection.

The study documented the dominant HIV risk factors among clients (Figure 2). Overall, 75% (n=1,001) of eligible clients were exposed to 3 or more risk factors. Unprotected vaginal or anal sex was the dominant risk factor (53%, n=707). Slightly more than a quarter (27%, n=360) of the clients who declined to start PrEP had a sexual partner with 1 or more HIV risk factors. Only 7% of the clients had a sexual partner who was HIV positive and not on ART for less than 6 months. Five percent (5%) had taken post-exposure prophylaxis following potential exposure to HIV.

#### Barriers to Uptake of Pre-Exposure Prophylaxis

**FIGURE 3:**
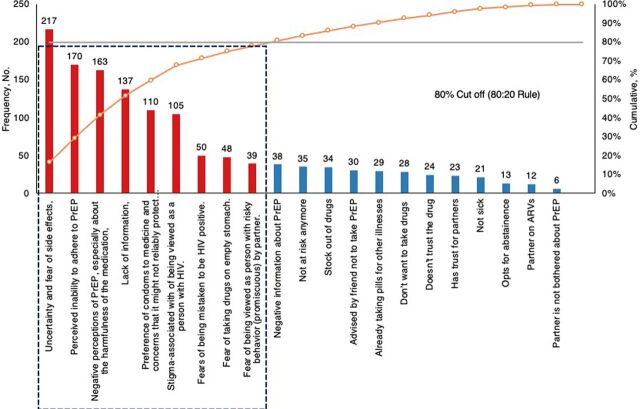
Main Barriers to Starting PrEP Among Eligible Clients for PrEP Abbreviations: ARV, antiretroviral; PrEP, pre-exposure prophylaxis.

The Pareto chart (Figure 3) shows the main barriers and contributing factors affecting the initiation of PrEP, which included uncertainty and fear of side effects, perceived inability to adhere to PrEP, and negative perceptions of PrEP. The emerging factors are supported by quotations that best represent the range of ideas voiced by the clients. The quotations were carefully edited without altering the meaning.

- **Uncertainty and fear of side effects**: “I was told that these drugs make a woman infertile,” or “I don’t want the drugs which reduce on my sexual appetite.”
- **Perceived inability to adhere to PrEP**: “I can’t take drugs everyday,” “I am not sure that I can take these drugs everyday.”
- **Negative perceptions of PrEP**: “The drug causes dryness during sex,” or “I fear PrEP will remove my pregnancy.”
- **Lack of information**: Underlying the rejection to start PrEP was lack of adequate information about PrEP, which explains the misconceptions around PrEP. “I don’t have enough information about PrEP,” or “I did not understand well how PrEP works.”
- **Preference of condoms to medicine and concerns that it might not reliably protect against HIV**: The clients believed more in the protective potential of condoms compared to PrEP and preferred using condoms. “I use condoms consistently [and] therefore don’t need to take those drugs,” or “I would rather use condoms whenever I am to have sex than take those drugs.”
- **Stigma associated with fears of being perceived to be HIV positive**: “Swallowing this drug is like you are HIV positive [and] this is what people will say,” “I do not need to take these drugs because am not sick.”
- **Fear of taking drugs on empty stomach**: The rejection to start PrEP can be attributed to lack of food. Clients perceived serious side effects if they took PrEP on an empty stomach. “I don’t have food to take these drugs and you can’t take them on an empty stomach,” “Those drugs are strong and require taking a lot of food which I don’t have.”
- **Fear of being perceived by partners as promiscuous**: The uptake of PrEP was limited by the fears of perceived promiscuity by the partner. “I don’t want my partners to see me taking these drugs because they will know that I go sleeping with everyone,” “I fear my partner will know that I am cheating on them with many other people and that is why am taking the drugs.”

#### Barriers to Continuity on Pre-Exposure Prophylaxis

**FIGURE 4:**
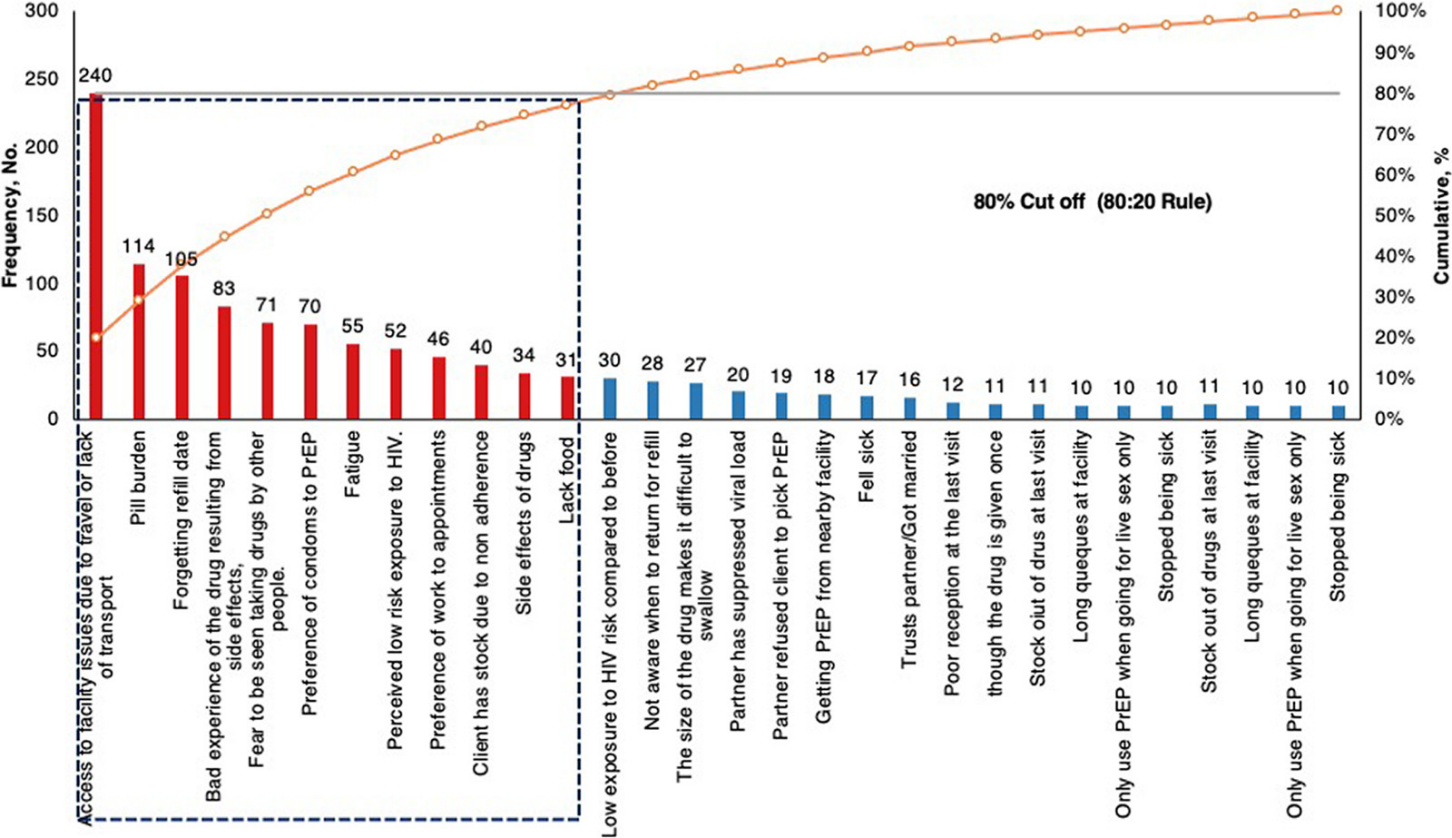
Main Barriers to Continuity on PrEP Among Clients Initiated on PrEP Abbreviation: PrEP, pre-exposure prophylaxis.

Clients who missed their appointments/refills were asked about the main reason why they missed their appointments or did not pick up drugs (Figure 4).

- **Lack of access to the facility due to travel or lack of transport:** Missing appointments for PrEP was driven by the lack of transport to the facility or because a client had traveled from their place of residence, which made it difficult to access the facility for their appointments. “I did not have money for transport to come to the facility,” “My work involves moving a lot and even right now am far away from home.”
- **Bad experience of the drug due to side effects**: Side effects were a main reason for missing appointments and not picking up PrEP pills. Drowsiness, headache, dizziness, vomiting, fatigue, nausea, and loss of appetite were mostly reported, despite some clients reporting recommended strategies such as eating before medication. “I stopped taking the drugs because I feel a lot of dizziness after taking them,” “The drugs make me feel bad when I take them. I feel bad like wanting to vomit each time I take them.”
- **Fear of being seen taking drugs by other people**: Clients feared taking drugs known for treating people living with HIV. The stigma arising from these fears kept clients from appearing at the facility.
- **Forgetting the refill date**: Clients reported forgetting their PrEP refill date and going about their routine schedules as if nothing happened. Forgetting appointments was largely attributed to busy work schedules.
- **Perceived low risk of exposure to HIV**: Clients who perceived themselves to be at low risk of HIV missed their appointment or picking up drugs at the facility. Clients perceived themselves to be at low risk of exposure to HIV when previously predisposing factors to HIV were no longer applicable (e.g., a client previously engaged in multiple sexual partnerships but had settled to 1 partner). “I used to sleep with many people. But now am not doing it very often,” “I now use condoms when am having sex with different people because I don’t know their HIV status.”
- **Preference for condoms**: Another reason for missing appointments was the preference for condoms over oral PrEP.
- **Pill burden and fatigue**: Clients expressed dislike for pills and concerns about the size of PrEP pills, arguing that they cannot comply with the daily therapy.
- **Lack of food**: Like their counterparts who declined to start PrEP, clients who missed appointments expressed the lack of food, which would exacerbate the much-feared/experienced side effects of the pills.

### Pre-Exposure Prophylaxis Collaborative

**FIGURE 5:**
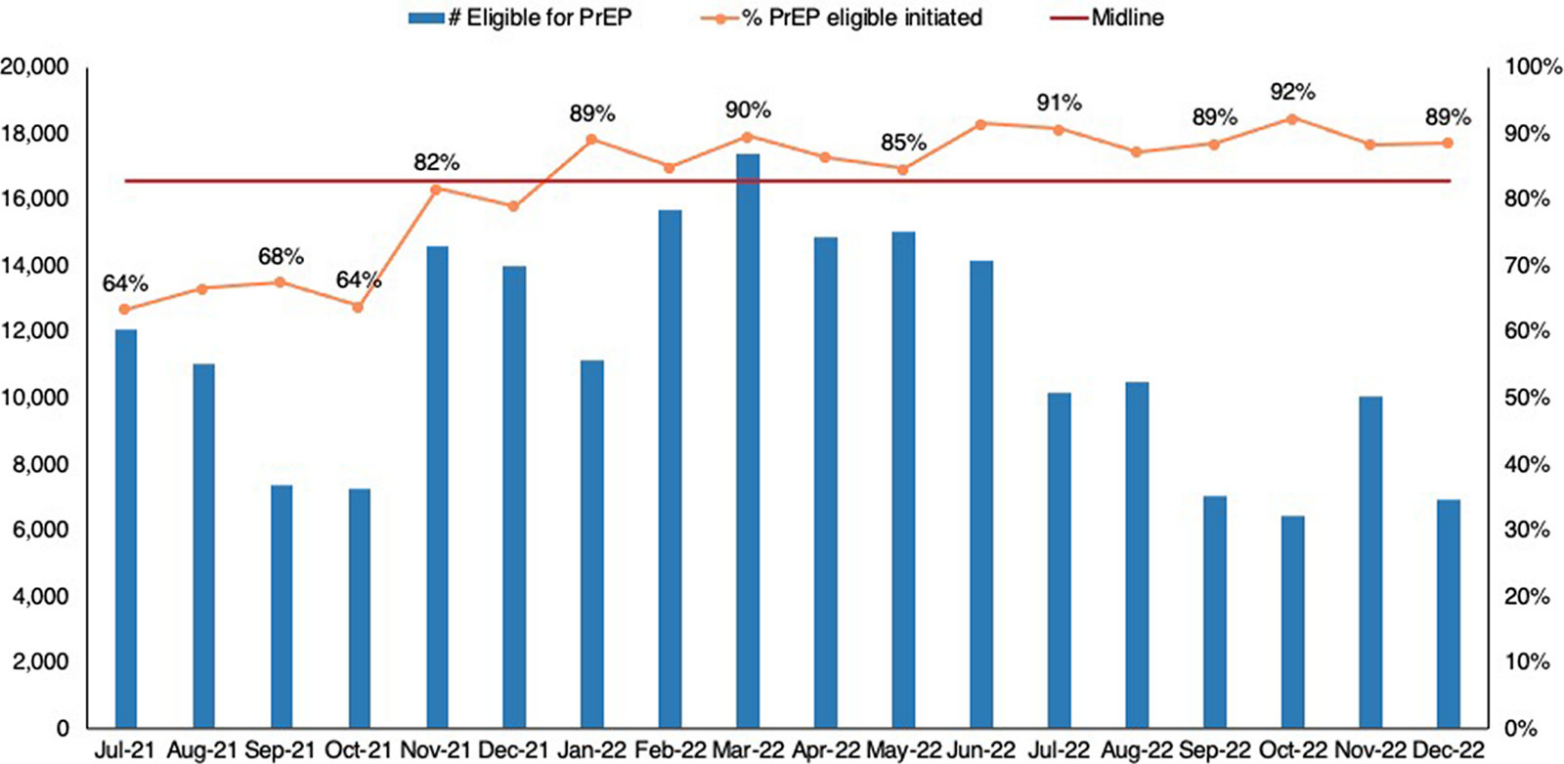
Percentage of Eligible Clients Who Were Initiated on PrEP Abbreviation: PrEP, pre-exposure prophylaxis.

**FIGURE 6:**
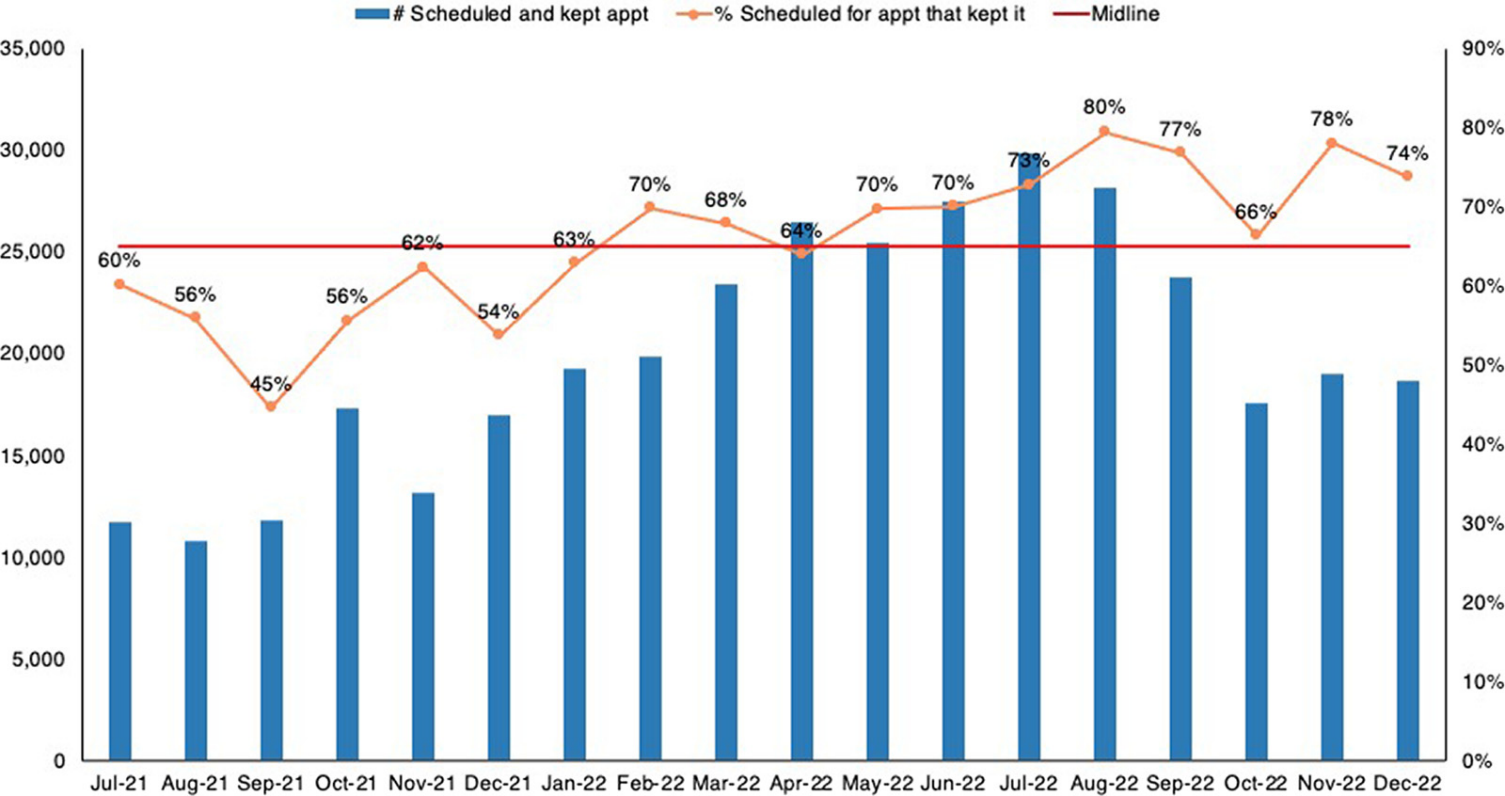
Percentage of PrEP Clients Who Were Scheduled for an Appointment and Kept It Abbreviation: PrEP, pre-exposure prophylaxis.

There were statistically significant shifts in the PrEP cascade implied by more than 4 data points above the median. Initiation on PrEP improved from 64% in July 2021 to 89% in December 2022 (Figure 5), while keeping appointments improved from 60% to 74% (Figure 6).

**FIGURE 7:**
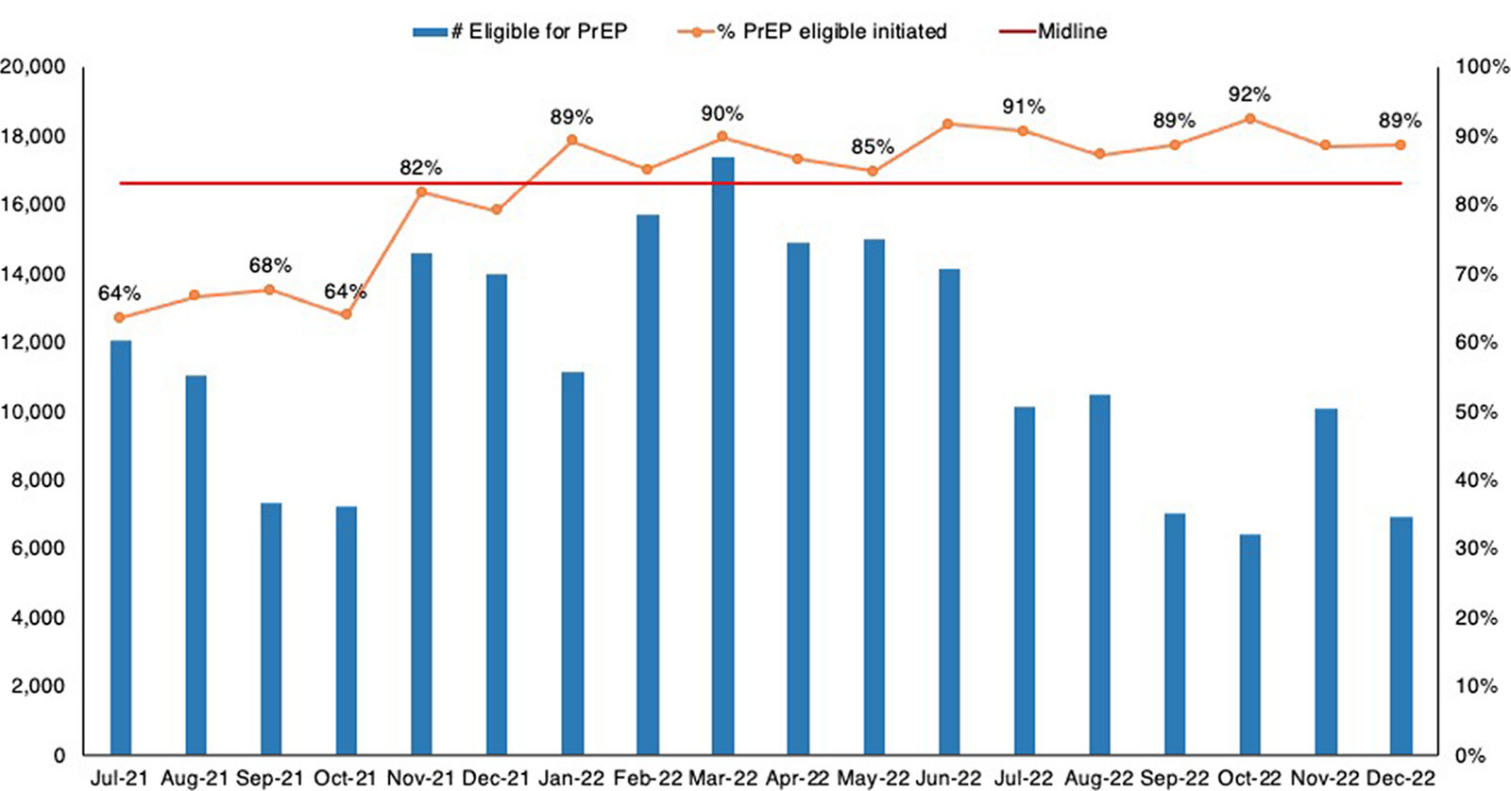
Percentage of PrEP Clients Who Missed Appointments, Were Followed Up, and With Documented Outcomes Abbreviation: PrEP, pre-exposure prophylaxis.

**FIGURE 8:**
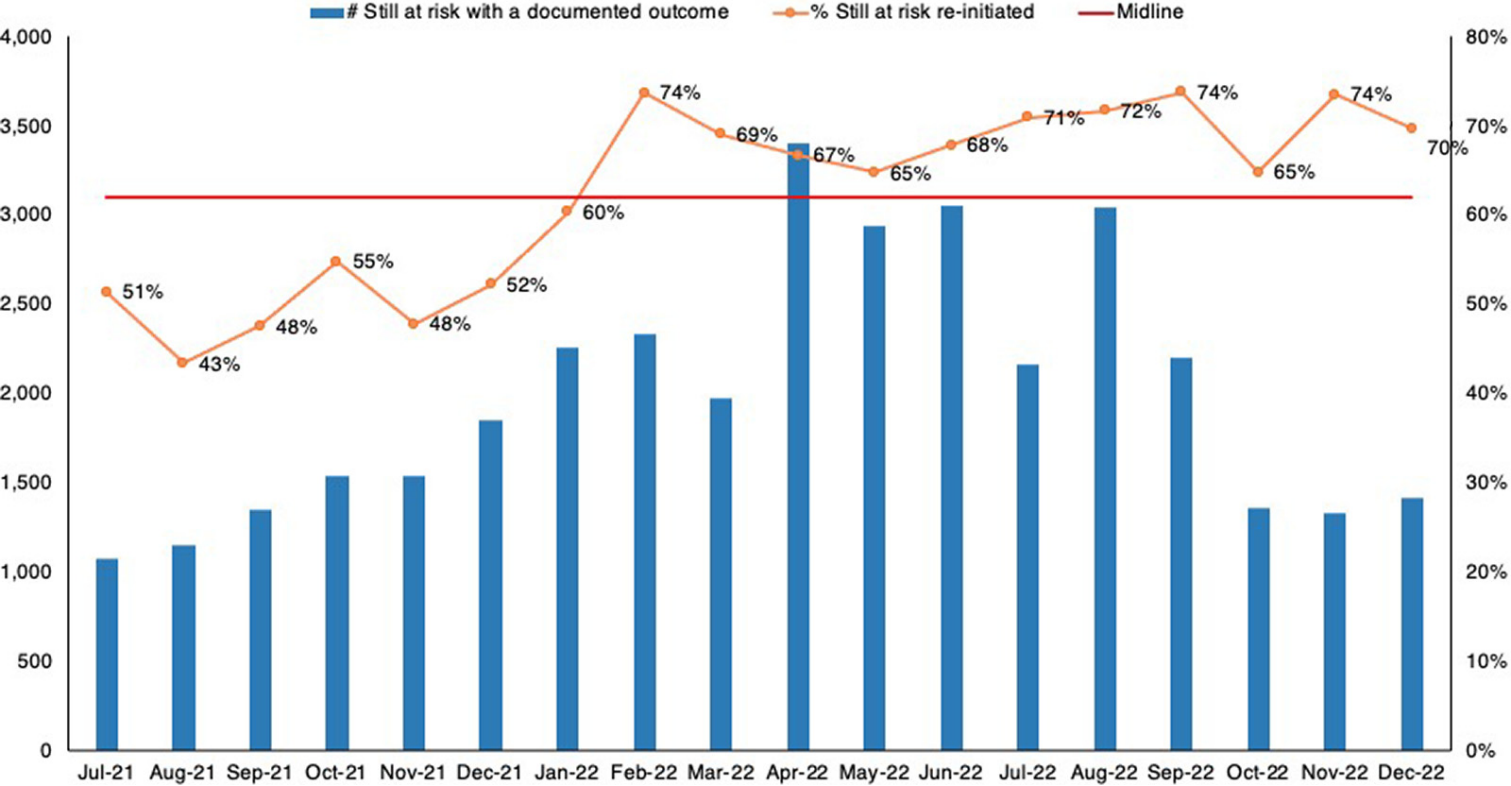
Percentage of Clients Followed Up, Still at Risk, and Reinitiated on PrEP Abbreviation: PrEP, pre-exposure prophylaxis.

Documented follow-up of missed appointments improved from 31% in July 2021 to 61% in December 2022 (Figure 7), while reinitiation on PrEP of clients still at risk improved from 51% to 70% (Figure 8). The improvements in the PrEP cascade are attributed to 1 or more of the tested changes for improving initiation and continuity on PrEP contained in the Supplement.

### Interventions Associated With Improvements in Initiation and Continuity of Pre-Exposure Prophylaxis

To tackle the barriers to PrEP initiation and continuity, facility teams implemented 1 or more of the change ideas (Table 2), tailored to address specific challenges uncovered through RCA findings and further elaborated upon in the Supplement.

## DISCUSSION

This article describes a 3-tier approach for determining barriers to the uptake of PrEP and how to address gaps and improve service delivery processes. Our gap analysis identified persons initiating tenofovir/emtricitabine for the PrEP indication in Uganda between 2019 and 2020. Understanding the gap and root causes behind these gaps and realigning PrEP resources is needed for effective PrEP programming. The novelty of the RCA study lies in the fact that it collected information on different perspectives that determine acceptability and continuity. The study found that there are diverse barriers to the uptake of PrEP, such as fear of side effects, misconceptions, low perception of risk, and perceived inability to adhere. These barriers and many others have been documented in other studies in similar settings in Zimbabwe.^18^

Understanding the gap and root causes behind gaps in PrEP uptake and continuity and realigning PrEP resources is needed for effective PrEP programming.

Our study found that individuals declined PrEP because they did not perceive themselves to be at high risk of HIV. The effect of risk perception on PrEP uptake has been documented in other studies and shows that individuals will take PrEP when they perceive themselves to be at a high risk of HIV.^19^ Earlier PrEP studies have found that uptake and continuity were higher among persons who exhibited a greater risk of acquiring HIV, such as unprotected sex in multiple sexual encounters.^20^

We identified knowledge and information gaps on PrEP as a barrier to uptake and continuity on PrEP. These findings are consistent with results from a study that explored barriers and facilitators of PrEP uptake among young people in Zimbabwe and South Africa.^14^ Because PrEP is an antiretroviral medication, screening, prescription, and health education is done by a health provider. However, human resource constraints within our context and similar contexts could undermine the provision of adequate information for informed decision-making by the client to start PrEP. Empowering peers to create awareness about PrEP could go a long way in demand generation for PrEP uptake.

In contrast, poor access to the health facility due to distant locations was a major barrier to continuity in our study. Accessibility to facility concerns has also been reported among the high-risk population in Zimbabwe.^14^

Known barriers to uptake and continuity on PrEP require interventions mapped to each unique barrier. Various treatment strategies have been employed in sub-Saharan Africa to address continuity and stigma. Among these strategies is the involvement of peers in supporting continuity.^21^ A PrEP collaborative was implemented to address barriers to PrEP uptake and continuity on PrEP. The intervention involved community-based health workers or peers in refilling clients and counseling on PrEP (Supplement). In Tanzania and Zambia, community-based health workers or peers were involved in PrEP service delivery, counseling, and adherence support for PrEP users.^22^ In Thailand, peer lay providers improved uptake through screening for eligibility and provision of PrEP regimens to users.^23^ Thus, we used nonphysicians, such as peers, in the delivery of PrEP refills and adherence support for PrEP users.

To address gaps in continuity on PrEP for persons at risk of HIV, the PrEP collaborative used different approaches (Supplement), including the use of a multimonth dispensing approach in which clients were given a refill of up to 3 months. This strategy is contained in the national HIV treatment strategies^1^ and has been adapted for HIV patients in most high-prevalence countries.^21^ In the PrEP collaborative, multimonth dispensing was adapted, especially among clients who could readily access facilities because of their social mobility, lack of transport, fear, and stigma-related factors established by the RCA. The improvements in the PrEP cascade were associated with the implementation of 1 or more of these interventions.

The study and the intervention to address barriers focused on high-risk groups eligible for PrEP. The consolidated guidelines for the prevention and treatment of HIV and AIDS in Uganda define high-risk groups to include population categories of female sex workers, fisher folks, adolescent girls, and young women. Similar groups have been defined as high-risk populations by other studies.^1^^–^^3^ In a PrEP program, individuals in populations at high risk of HIV should be assessed to determine whether they individually have risk factors warranting PrEP use and whether they are free of barriers that would influence their use of PrEP.^19^ In tandem with these studies, our study documented the barriers affecting the uptake and continuity on PrEP and an intervention to address them through a PrEP collaborative under the 3-tier approach.

### Lessons Learned

Using a 3-tier approach involving a comprehensive assessment of barriers at multiple levels enabled a thorough understanding of the complex factors influencing PrEP utilization.

Using a 3-tier approach involving a comprehensive assessment of barriers at multiple levels, enabled a thorough understanding of the complex factors influencing PrEP utilization.

The study emphasizes the importance of tailored interventions at multiple levels (individual, community, and health facility) to address sociocultural, behavioral, structural, and knowledge factors influencing PrEP uptake and continuity. Notably, the involvement of peers is identified as critical in supporting PrEP demand creation, initiation, and adherence among high-risk populations.

The significant improvements observed in PrEP uptake and continuity following the collaborative intervention underscore the value of integrating QI approaches into PrEP programs.

### Recommendations

The 3-tier approach is a stepwise approach to identifying and addressing gaps and barriers to PrEP uptake and should be adopted in similar jurisdictions and contexts.

Implementation strategies involving differentiated PrEP service delivery (e.g., a person-centered approach to PrEP service delivery), using more inclusive messaging, and further integrating PrEP within health care services may help to reduce PrEP stigma and ultimately increase PrEP use.

Our intervention underscores the vital importance of peers in every stage of the PrEP process, from generating demand to ensuring adherence among high-risk populations. Therefore, PrEP initiatives should actively use peer social networks and influencers.

PrEP delivery to high-risk populations should seek to identify delivery mechanisms that are appealing to different groups while also mitigating access barriers, such as stigma, privacy, convenience, time, and accessibility factors.

On this basis, we offer the following recommendations about how to improve continuation and address barriers to initiation.

- Providers and counselors should have appropriate and well-packaged information on PrEP to provide prescriptions with adequate counseling to improve acceptability of PrEP.
- Multimonth dispensing should be used, especially for clients not likely to meet their appointment schedule.
- Nonphysicians should be enabled to counsel, prescribe PrEP, and follow up with clients who are continuously at risk of HIV.
- PrEP should be integrated with other services to diminish the stigma associated with PrEP. PrEP programming should be integrated with existing prevention programs targeting high populations at risk of HIV. Multilevel interventions that target different levels of teams of providers, peers, and coaches should be used to identify, categorize, and analyze barriers to PrEP uptake.

### Limitations

The RCA was a cross-sectional analysis of barriers to uptake of PrEP and keeping appointments; therefore, it could not capture changes in barriers over time. Secondly, the dynamic implementation of initiatives to improve PrEP service provisions is likely to continually shape the types of recommendations coming from such initiatives. Despite these limitations, this study and interventions provide important insights for PrEP implementation. Our study and interventions focused broadly on the perspectives of clients through RCA and health care providers through the PrEP collaborative. Therefore, the data produced and interpreted for this article reflect the different key stakeholders of any PrEP program.

## CONCLUSION

There are multiple barriers to PrEP initiation and continuation, such as fear of side effects, perceived inability to take daily pills, perceived low HIV risk, stigma, and preference for condoms, among others. The use of peers, appointment tracking, and pre-appointment reminders are among other high-impact interventions that greatly improved PrEP uptake and continuity. The 3-tier approach to PrEP implementation of analyzing gaps, identifying individual barriers to initiation and continuity on PrEP, and implementing high-impact interventions to address gaps in the processes of care is a multilevel intervention to achieve effective PrEP implementation.

## Supplementary Material

GHSP-D-23-00229-supplement.pdf

## Notes

**Peer Reviewed**

**First published online:** March 12, 2024.
