# Supplementary material for: Applying a Three-Tier Approach to Address Gaps in Oral Pre-Exposure Prophylaxis Uptake and Continuity in Uganda: A Mixed Methods Approach
Source: Glob Health Sci Pract. 2024 Apr 29;12(2):e2300229. doi: 10.9745/GHSP-D-23-00229 (PMC11057795; doi:10.9745/GHSP-D-23-00229)
Supplement: GHSP-D-23-00229-supplement.pdf [file GHSP-D-23-00229-supplement.pdf]

**Supplement to**: Sensalire S, Nkolo A, Ssali JN, Muhire M, Muhwezi A, Kadama H. Applying a 3-tier approach to address gaps in oral pre-exposure prophylaxis uptake and continuity in Uganda: a mixed methods approach. *Glob Health Sci Pract*. 2024;12(2):e2300229. https://doi.org/10.9745/GHSP-D-23-00229

## Supplement: Change package for improving PrEP initiation and continuity

| Specific problem being addressed | Change tested | Evidence of successful change and site(s) that implemented changes | How exactly was the change tested/implemented (who, where, when, how |
|---|---|---|---|
| **Best practices for improving PrEP initiation** | | | |
| PrEP tools do not have provision for recording PrEP screening | Improvised PrEP screening column in the: HTS, ANC, PNC registers to record screening as P-SCN and Eligible as E-SCN | Improved from: 70-95% (Bukomero HC IV) 70-93% (Jinja RRH) 0-83% (Sigulu HC III) | ▪ Use a ruler and draw 2 lines at the extreme end of the page of the registers (HTS, ANC and PNC)<br>▪ 1st line is labeled "screened" and 2nd line is labeled "eligible".<br>▪ Record Y for YES if screened and N for NO if not screened. For those screened record Y for YES if eligible and N for NO if not eligible. |
| Low screening due to low utilization of the PrEP screening tool | Attach PrEP screening tool to every HTS client card before screening is done | Improved from: 70-93% (Jinja RRH) 53-81% (Ngai HC III) | ▪ Assign a linkage facilitator to attach PrEP screening tool to the HTS client cards<br>▪ Make the screening tools available at all entry points<br>▪ Assign a focal person to review utilization of the PrEP screening tools daily and address any gaps identified accordingly. |
| No specific person to screen for PrEP eligibility at the HTS point of care. | Assign a specific person to screen for PrEP at all HTS point of care | Improved from: 44-58% (Buwenge HC IV) | ▪ Formed a team to perform PrEP screening.<br>▪ Share the screening criteria.<br>▪ Conduct coaching on how to screen for PrEP eligibility.<br>▪ Display the screening criteria at the screening points in the facility |
| PrEP screening was not done at all entry points | Conduct PrEP screening during other activities of the facility such as YCC, ANC, VMMC | Improved from: 53-81% (Ngai HC III) | ▪ Hold a WIT meeting.<br>▪ Agree on what need to be done to have PrEP as part of the activities in other departments.<br>▪ Distribute PrEP screening tools to all relevant departments to start using them.<br>▪ Conduct a CME on how to screen for PrEP eligibility. |
| Low PrEP initiation at hot spots | Attach peers to specific hot spots to sensitize clients on PrEP | Improved from: 51-91% (Awach HC IV), 94-100% (Rubaare HC IV), 28-100% (Bison HC III), 93-100% (Gulu RRH) | ▪ Identify peers at hot spots<br>▪ Orient them on PrEP<br>▪ Peers identify additional peers and orient them on PrEP<br>▪ Refer those that need PrEP to the facility |
| Lack of awareness about PrEP | Attach satisfied PrEP users -peer outreach workers to do group and individual education at facility and community | Improved from: 91-100% (Kamukira HC IV) | ▪ Identify satisfied PrEP users from among clients at various hot spots-select most influential peer from among the satisfied PrEP users<br>▪ Orient them on counselling on PrEP.<br>▪ Attach them to hot spots<br>▪ Develop a schedule for each hot spot and communicate to the peer |
| Incomplete documentation of PrEP registers | Form and task a team to review PrEP records for completeness | Improved from:45-80% (Buwama HC III) | ▪ A group of competent staff is formed<br>▪ Tasked to review PrEP gaps in documentation and report to responsible staff |

**Supplement to**: Sensalire S, Nkolo A, Ssali JN, Muhire M, Muhwezi A, Kadama H. Applying a 3-tier approach to address gaps in oral pre-exposure prophylaxis uptake and continuity in Uganda: a mixed methods approach. *Glob Health Sci Pract*. 2024;12(2):e2300229. https://doi.org/10.9745/GHSP-D-23-00229

| Specific problem being addressed | Change tested | Evidence of successful change and site(s) that implemented changes | How exactly was the change tested/implemented (who, where, when, how |
|---|---|---|---|
| Low uptake of PrEP | Assign a focal person to PrEP activities at the facility | Improved from 73-100% (Rakai Hospital) | ▪ A skilled and willing staff voluntarily accepted to take over as a PrEP focal person during a staff meeting |
| | Identify skilled and willing staff at entry points to take charge of PrEP initiation | Improved from 40-68% (Kakiri HCIV) | ▪ Skilled and willing staff from all entry point are identified and appointed to take charge of PrEP initiation in their departments |
| Lack of a system to track clients due for appointment | Use of SMS/phone calls as reminders for clients due for appointment | Improved from: 58-83% (Bukuku HC IV),15-74% (KIHEFO HC III), 69-100% (Atiak HC), 83-85% (Nakasongola HC IV) 23-100% (Mukono hospital) 32-61% (TASO Rukungiri) 58-87% (Batambogwe HC III) | ▪ Ask for clients' telephone numbers during enrollment<br>▪ Dial the number to confirm it is the right number<br>▪ Send SMS prior to the appointment date for those clients due<br>▪ Check the appointment register and line list clients due for appointment from the PrEP records- appointment, PrEP registers, client facility card, and the KP/ PrEP tracker<br>▪ Call clients to confirm availability for the appointment<br>▪ Reschedule those not available on the due date<br>▪ Transfer clients who missed appointments into the missed appointment register |
| Incomplete clients record in some PrEP tools | Develop and display a check list outlining tools required by health workers to complete during PrEP initiation | Improved from: 76-91% (Kamukira HC III) | ▪ Develop a check list of all tools required for PrEP initiation<br>▪ Orient staff on the tools<br>▪ Display the check list at places of PrEP initiation |
| | Conducted targeted CME to new staff/ departmental representatives from all entry points on PrEP initiation | Improved from: 62-98% (Rakai Hospital) | ▪ Identify new staff in each department<br>▪ Communicate to the new staff through whats-up platform about a CME<br>▪ Invite the new staff to attend the CME<br>▪ Select the presenter of the CME<br>▪ Develop action points and way forward from the CME<br>▪ Schedule weekly CMEs for various department heads<br>▪ Task the heads to disseminate PrEP information to other staff |
| Long distance for clients to access services | Conduct targeted outreaches | Improved from: 45-66% (Buwama HC III) | ▪ Map areas with underserved such as hot spots<br>▪ Schedule outreaches for such areas assign staff to such places to conduct PrEP initiation |
| **Change ideas for improving appointment keeping** | | | |
| Lack of a system to track clients due for appointment | Use of SMS/phone calls as reminders for clients due for appointment | Improved from: 58-83% (Bukuku HC IV),15-74% (KIHEFO HC III), | ▪ Ask for clients' telephone numbers during enrollment<br>▪ Dial the number to confirm it is the right number |

**Supplement to**: Sensalire S, Nkolo A, Ssali JN, Muhire M, Muhwezi A, Kadama H. Applying a 3-tier approach to address gaps in oral pre-exposure prophylaxis uptake and continuity in Uganda: a mixed methods approach. *Glob Health Sci Pract*. 2024;12(2):e2300229. https://doi.org/10.9745/GHSP-D-23-00229

| Specific problem being addressed | Change tested | Evidence of successful change and site(s) that implemented changes | How exactly was the change tested/implemented (who, where, when, how |
|---|---|---|---|
| | | 69-100% (Atiak HC) 83-85% (Nakasongola HC IV), 23-100% (Mukono hospital) 32-61% (TASO Rukungiri) 58-87% (Batambogwe HC III) | ▪ Send SMS prior to the appointment date for those clients due<br>▪ Check the appointment register and line list clients due for appointment from the PrEP records- appointment, PrEP registers, client facility card, and the KP/ PrEP tracker<br>▪ Call clients to confirm availability for the appointment<br>▪ Reschedule those not available on the due date<br>▪ Transfer clients who missed appointments into the missed appointment register |
| Client does not show up for appointment | Attach clients due for appointment to peers | Improved from: 58-83% at Nakasongola HC IV, 63-100% at Anyeke HC IV 20-58% at Batambogwe HC III | ▪ After line lists, map areas where clients due for appointment come from<br>▪ Identify peers from the mapped areas<br>▪ Attach clients to peers from the same area for follow up and remind them to show up for their appointments<br>▪ Conduct a meeting to review challenges with appointment keeping<br>▪ Review files and arrange them by hot spot<br>▪ Attach clients to peers by hot spot and location<br>▪ Define roles of the peers in relation to follow up<br>▪ Seek consent from clients before attaching them to peers |
| Health workers do not know clients' locations for follow up | Develop a locator form and update locations whenever they return to the clinic | Improved from: 33-100% (Mukono hospital) | ▪ Develop a one-page form to capture client details, location, and phone contact<br>▪ Print multiple copies<br>▪ Attach the copies to the client file<br>▪ Complete the form at every encounter with a client |
| Transport challenges by clients | Prioritize hot spots with high numbers of clients to deliver PrEP refills to them | Improved from: 56-87% (Baitambogwe HC III) 48-85% (KIHEFO HC III) | ▪ Identify hot spots with high numbers of clients<br>▪ Line list clients in those hot spots<br>▪ Call the clients before visiting their area to schedule appointments<br>▪ Pre-pack drugs and other commodities that they will need and deliver them to the hot spots |
| Client stigma while at the facility | Avail PrEP refills at DICs | Improved from: • 48-74% (Nakasongola HC IV and Kyenjojo hospital | ▪ Attach trained peers at the DICs to conduct PrEP refills<br>▪ Conduct outreaches for PrEP refills to DICs |
| Clients experience long wait times at the clinic | Conduct PrEP refills during outreaches of other activities in the community | Improved from:15-94% (KIHEFO HC III), 63-100% (Anyeke HC IV) | ▪ Take note of the outreach schedule.<br>▪ Align PrEP refills to the outreach schedule<br>▪ Mobilize clients to gather at the outreach post on the due date<br>▪ Carry refills during the outreach<br>▪ Provide the refills to the clients who gathered |
| | Set up a one-stop center at the facility | Improved from: 23-100% (Mukono hospital) | ▪ In a meeting, agree to provide PrEP at designated points of care<br>▪ Assign a focal person at each service point<br>▪ Set up an inter-referral system for PrEP clients<br>▪ Equip the service delivery points with medicines and logistics to provide PrEP |

**Supplement to**: Sensalire S, Nkolo A, Ssali JN, Muhire M, Muhwezi A, Kadama H. Applying a 3-tier approach to address gaps in oral pre-exposure prophylaxis uptake and continuity in Uganda: a mixed methods approach. *Glob Health Sci Pract*. 2024;12(2):e2300229. https://doi.org/10.9745/GHSP-D-23-00229

| Specific problem being addressed | Change tested | Evidence of successful change and site(s) that implemented changes | How exactly was the change tested/implemented (who, where, when, how |
|---|---|---|---|
| Patients' illiteracy on PrEP | Translate IEC materials in local language | Improved from: 58-83% (Bukuuku HC IV) | ▪ Translate available IEC materials into the local language<br>▪ Print the materials<br>▪ Display them where patients can access them |
| **Improving documentation of outcomes** | | | |
| Lack of documentation of follow up outcomes | Develop and update the locator form | Improved from 58-81% (Ngoma HCIV | ▪ At clinic visit, ask all client to share correct phone contacts<br>▪ Update them whenever they have changes |
| | Generate a line list of missed appointments | Improved from 6-40% (Wakiso HCIV | ▪ Use a KP tracker, PrEP register, facility client form to generate a line list |
| | Make a phone call to clients who missed appointments | Improved from 27-68% (MAAPI Clinic | ▪ At the end of the clinic day, generate a list of clients that missed appointments<br>▪ Distribute the list to peers and counsellors to make phone calls to follow up with those clients |
| Lack of responsible person to track missed appointments | Assign a health worker and peers to physically follow up missed appointments | Improved from 0-100% (Buyanja HC III), 0-79% (Bufunda HCIII, 90-100% (Kakabale HC III), 56-81% (Ngoma HCIV) | ▪ Develop a schedule for health workers and peers to do physical follow up of missed appointments at different hot spots<br>▪ Record outcomes in the appointment register upon return<br>▪ During a QI meeting, roles are discussed and allocated to different staff |
| Knowledge gap for health workers on filling the appointment register | Hands on orientation on filling the appointment register | Improved from: 36-57% (Ngoma HC IV) | ▪ Demonstrate to staff how to update the appointment register with outcomes as collected from the clients |
| Forgetting to update follow up outcomes | Same day update of follow up outcomes | Improved from:40-50% (Wakiso HC IV) | ▪ At the end of the clinic day, assigned staff update the respective tools with the outcomes that were collected from the clients immediately after each phone call |
| **Improving PrEP re-initiation of missed appointment still at risk** | | | |
| Long distance | Community PrEP drug refills | Improved from: 35-65% (Bwindi community hospital) 46-76% (Jinja RRH) | ▪ Line list clients that missed appointments and were followed up and are still at risk<br>▪ Identify staff to go for the outreaches<br>▪ Map location of the clients<br>▪ Ask the peers to mobilize the clients<br>▪ Do PrEP re-initiation in the community during outreaches |
| Re- initiated clients are not recorded | Assign a health work to update clients that are re-initiated on PrEP | Improved from: 50-80% (Kibaale HC IV) | ▪ PrEP focal person identifies a staff and is allocated to update all clients that have been re-initiated on PrEP<br>▪ Hands on orientation on completing the register is done<br>▪ Updates are done immediately after re-initiation |

**Supplement to**: Sensalire S, Nkolo A, Ssali JN, Muhire M, Muhwezi A, Kadama H. Applying a 3-tier approach to address gaps in oral pre-exposure prophylaxis uptake and continuity in Uganda: a mixed methods approach. *Glob Health Sci Pract*. 2024;12(2):e2300229. https://doi.org/10.9745/GHSP-D-23-00229
